# Atlas‐based assessment of hypomyelination: Quantitative MRI in Pelizaeus‐Merzbacher disease

**DOI:** 10.1002/hbm.70014

**Published:** 2024-09-04

**Authors:** Caroline Köhler, Paul Kuntke, Prativa Sahoo, Hannes Wahl, Sean C. L. Deoni, Jutta Gärtner, Steffi Dreha‐Kulaczewski, Hagen H. Kitzler

**Affiliations:** ^1^ Institute of Diagnostic and Interventional Neuroradiology, Faculty of Medicine and University Hospital Carl Gustav Carus Technische Universität Dresden Dresden Germany; ^2^ Department of Pediatrics and Adolescent Medicine University Medical Center Göttingen Göttingen Germany; ^3^ Advanced Baby Imaging Lab, School of Engineering Brown University Providence Rhode Island USA; ^4^ Maternal, Newborn, and Child Health Discovery and Tools Bill and Melinda Gates Foundation Seattle Washington USA

**Keywords:** hypomyelinating leukodystrophy, magnetic resonance imaging, medical image registration, neuroimaging, Pelizaeus‐Merzbacher disease, white matter disorder

## Abstract

Pelizaeus‐Merzbacher disease (PMD) is a rare childhood hypomyelinating leukodystrophy. Quantification of the pronounced myelin deficit and delineation of subtle myelination processes are of high clinical interest. Quantitative magnetic resonance imaging (qMRI) techniques can provide in vivo insights into myelination status, its spatial distribution, and dynamics during brain maturation. They may serve as potential biomarkers to assess the efficacy of myelin‐modulating therapies. However, registration techniques for image quantification and statistical comparison of affected pediatric brains, especially those of low or deviant image tissue contrast, with healthy controls are not yet established. This study aimed first to develop and compare postprocessing pipelines for atlas‐based quantification of qMRI data in pediatric patients with PMD and evaluate their registration accuracy. Second, to apply an optimized pipeline to investigate spatial myelin deficiency using myelin water imaging (MWI) data from patients with PMD and healthy controls. This retrospective single‐center study included five patients with PMD (mean age, 6 years ± 3.8) who underwent conventional brain MRI and diffusion tensor imaging (DTI), with MWI data available for a subset of patients. Three methods of registering PMD images to a pediatric template were investigated. These were based on (a) T1‐weighted (T1w) images, (b) fractional anisotropy (FA) maps, and (c) a combination of T1w, T2‐weighted, and FA images in a multimodal approach. Registration accuracy was determined by visual inspection and calculated using the structural similarity index method (SSIM). SSIM values for the registration approaches were compared using a *t* test. Myelin water fraction (MWF) was quantified from MWI data as an assessment of relative myelination. Mean MWF was obtained from two PMDs (mean age, 3.1 years ± 0.3) within four major white matter (WM) pathways of a pediatric atlas and compared to seven healthy controls (mean age, 3 years ± 0.2) using a Mann–Whitney *U* test. Our results show that visual registration accuracy estimation and computed SSIM were highest for FA‐based registration, followed by multimodal, and T1w‐based registration (SSIM_FA_ = 0.67 ± 0.04 vs. SSIM_multimodal_ = 0.60 ± 0.03 vs. SSIM_T1_ = 0.40 ± 0.14). Mean MWF of patients with PMD within the WM pathways was significantly lower than in healthy controls MWF_PMD_ = 0.0267 ± 0.021 vs. MWF_controls_ = 0.1299 ± 0.039. Specifically, MWF was measurable in brain structures known to be myelinated at birth (brainstem) or postnatally (projection fibers) but was scarcely detectable in other brain regions (commissural and association fibers). Taken together, our results indicate that registration accuracy was highest with an FA‐based registration pipeline, providing an alternative to conventional T1w‐based registration approaches in the case of hypomyelinating leukodystrophies missing normative intrinsic tissue contrasts. The applied atlas‐based analysis of MWF data revealed that the extent of spatial myelin deficiency in patients with PMD was most pronounced in commissural and association and to a lesser degree in brainstem and projection pathways.

## Graphical abstract

FA‐based template registration is superior to conventional T1w registration in patients with hypomyelinating Pelizaeus‐Merzbacher disease (PMD) and provides an alternative approach that enables atlas‐based assessment of myelination using quantitative magnetic resonance imaging in hypomyelinating disorders.

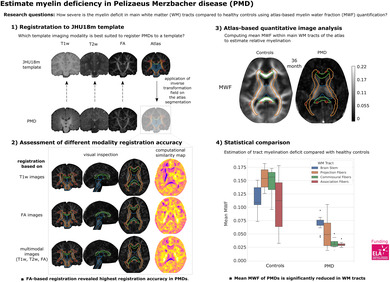

## 1 INTRODUCTION

Pelizaeus‐Merzbacher disease (PMD) is a rare hypomyelinating white matter (WM) disorder caused by a defect in the PLP1 gene that disturbs the formation of myelin (Garbern, 2007). Depending on the type of mutation, such as deletion, point mutation, duplication or triplication (Wolf et al., 2005), a wide range of myelin deficiency has been described (Koeppen & Robitaille, 2002; Laukka et al., 2016). The myelin deficit can cause a variety of neurological symptoms and very distinct developmental delays. In the classic course of the disease, patients characteristically present with nystagmus and extreme muscle hypertonia in infancy. Later on, they develop ataxia and leg spasticity. The developmental delays affect motor skills and cognition, particularly speech and communication difficulties (Golomb et al., 2004; Moore et al., 2023).

Hypomyelinating leukodystrophies (HLD) have been characterized in general by conventional magnetic resonance imaging (MRI), which allows qualitative estimation of the myelin deficiency. For example, Schiffmann and Van der Knaap, reported that if very little to no myelin is present within brain WM, the cerebral WM signal is lower than that of gray matter (GM) structures on T1‐weighted (T1w) images (Schiffmann & Van Der Knaap, 2009). As myelination increases, WM becomes iso‐ and then hyperintense to GM structures on T1w images. However, advanced quantitative MRI techniques (qMRI) might be more appropriate to determine brain myelination in patients with HLD (Pouwels et al., 2014) to capture spatial myelin deficits for monitoring the natural course of the disease. QMRI may provide a better characterization of the PMD phenotypes and may serve as a biomarker for myelin‐specific therapeutic efficacy in the future (Stellingwerff et al., 2023). Myelin water imaging (MWI), which allows the determination of the myelin water fraction (MWF), is currently considered to be highly specific for the quantification of myelin in vivo (MacKay et al., 2006). Further technical developments (Deoni et al., 2008) have enabled whole‐brain coverage in clinically acceptable scan times (Prasloski et al., 2012).

Considerable research has been conducted over the past decades to develop qMRI sequences to provide in vivo insights into brain microstructure and myelination. Little research has addressed MRI analysis pipelines for developing and evaluating diseased pediatric brains, particularly those with low or deviant image contrast compared to healthy controls. For this purpose, a template of healthy control participants with segmented brain structures (atlas), which serves as a region of interest (ROI), is transferred to the qMRI data by registration in the patient image space, allowing for quantitative image analysis and statistical comparison.

However, the lack of reliable registration methods for aligning pediatric brain imaging data with severely deviating WM/GM tissue contrast to a healthy brain template is a major problem for atlas‐based analyses. Template registration requires images with similar image contrast, which is typically performed on T1w images.

Groups developing MRI templates for children have suggested that fractional anisotropy (FA) images can guide registration in sparsely myelinated neonates with poor WM/GM T1w contrast (Huang et al., 2006; Oishi et al., 2011). Further, FA contrast of main WM tracts was found to be more stable than T1w WM contrast during brain myelination (Zhang et al., 2007) and therefore may serve as a better registration target image in hypomyelinated patients with PMD. Presumably, the inclusion of multimodal images in the registration process may provide additional information to render the registration process more accurate.

This study aimed to develop an optimized postprocessing pipeline for atlas‐based image evaluation of qMRI data obtained from patients with PMD. Three registration methods based on (a) T1w, (b) FA, or (c) a multimodal approach combining T1w, T2‐weighted (T2w), and FA images were implemented. Furthermore, the registration accuracy was estimated by visual inspection and structural similarity index (SSIM) calculation. In addition, the optimal registration approach was selected to analyze the MWF of patients with PMD in the main WM tracts of a pediatric atlas and compared to age‐matched controls to estimate spatial myelin deficiency.

## 2 METHODS

### 2.1 Patient cohort and healthy control dataset

Patients with PMD were recruited by a pediatric white matter disease center between 2014 and 2020. A total of five male pediatric patients with a genetically confirmed diagnosis of PMD were included in this study. The institutional ethical review board approved all study procedures. MRI visits were performed under sedation during an inpatient stay as part of the clinical evaluation. In addition to structural imaging, MWI data were available in two patients. Informed consent was obtained from the parents prior to the study. An additional MWI control dataset of seven male participants was used with permission to compare with the PMD subgroup (Deoni et al., 2012). Demographics are presented in Table 1.

**TABLE 1:** Demographics.

	No. of participants	Age (years) mean (min, max)
Healthy controls	7	3 (2.7, 3.1)
PMD‐registration dataset	5	6 (2.8, 11.2)
PMD‐MWI subset	2	3.1 (2.9, 3.3)

### 2.2 MRI data acquisition

A 3‐Tesla scanner (TrioTim, Siemens Healthineers, Erlangen, Germany) equipped with an 8‐channel radiofrequency head coil was used to acquire MRI data. 3D T1w, 3D T2w, MWI, and DTI with 64 gradient directions with two sets of b0 images with reversing phase encoding direction were acquired according to the study protocol (Table 2). The MWI technique (multi‐component driven equilibrium single pulse observation of T1/T2 [mcDESPOT]) was used, allowing the processing of high‐resolution whole‐brain MWF maps (Deoni et al., 2008). Following the mcDESPOT protocol, spoiled gradient recalled echo (SPGR) and balanced steady‐state free precession (bSSFP) sequences were acquired over a range of flip angles at constant TE and TR (Deoni et al., 2012). Further, inversion‐prepared (IR)‐SPGR was acquired to correct for transmit (B1) magnetic field inhomogeneity, and bSSFP data was acquired with 0° and additionally with 180° phase increments to correct for main (B0) field variations. The MRI data were acquired during a total scan time of approximately 37 min.

**TABLE 2:** MRI protocol.

	3D MPRAGE	3D TSE	DTI	SPGR	bSSFP	IR‐SPGR
TR, ms	2250	2900	7500	5.4	4.9	5.4
TE, ms	3.26	428	82	2.5	9.8	2.5
Flip angle, degrees	9	120	90	[3, 4, 5, 6, 7, 9, 13, 18]	[9, 12, 15, 18, 21, 28, 40, 55]	5
Inversion time, ms	900	‐	‐	‐		450
Resolution, mm	1 × 1 × 1	1 × 1 × 1	2 × 2 × 2	1.7 × 1.7 × 1.7	1.7 × 1.7 × 1.7	2.3 × 2.3 × 3.5
Gradient directions	‐	‐	64 at b = 1000s/mm^2^	‐	‐	‐
Phase cycling, degrees	‐	‐	‐	‐	[0, 180]	‐
Scan time, (min:s)	07:17	05:07	08:06	05:21	2× 04:57	01:14

Abbreviations: 3D T1‐weigthed MPRAGE, magnetization‐prepared rapid gradient echo; 3D T2‐weighted TSE, turbo spin echo; DTI, diffusion tensor imaging; SPGR, spoiled gradient echo; bSSFP, balanced steady‐state free precession; IR‐SPGR, inversion‐prepared spoiled gradient echo.

### 2.3 DTI processing

DTI data were first corrected for eddy‐current‐induced distortion using “topup” and “eddy” routines and then FA maps were derived using “dtifit” routine from the FMRIB's Software Library (FSL). The rigid transformation was used to align DTI images to the T1w image (reference image) within the session using the least diffusion‐weighted images (b0 images) as moving images. The transformation matrix was then applied to the FA maps.

### 2.4 Processing of myelin water fraction maps

The MWF computation was based on the algorithms previously described by Deoni and Kolind (Deoni & Kolind, 2015), but was implemented in a Nipype workflow (Neuroimaging in Python: Pipelines and Interfaces) (Gorgolewski et al., 2011) to manage and process the files of the mcDESPOT dataset. This enabled the use of multithreading and high‐performance computers. SPGR, IR‐SPGR, and bSSFP images were co‐registered with the high flip angle SPGR image (SPGRfa18). To obtain quantitative T1 and B1 field maps, the SPGR and IR‐SPGR scans were used for DESPOT1‐HIFI analysis (Deoni, 2007). T2 and B0 field maps were then calculated using DESPOT2 from the bSSFP data and the quantitative T1 and B1 maps. Finally, the B0 and B1 maps, as well as SPGR and bSSFP 0° and 180°, were used to estimate the MWF using stochastic region contraction by fitting the SPGR and bSSFP data to a three‐pool relaxation model of WM (Deoni et al., 2013). An affine image transformation was used to align the SPGRfa18 image to the T1w image (reference image) within the session, and the resulting transformation matrix was then applied to the MWF map.

### 2.5 Pediatric template

A pediatric template (https://cmrm.med.jhmi.edu/, JHU_Pediatric_SS_18Month (JHU18m)) was chosen that provided aligned T1w, T2w, and FA (multimodal) images obtained from a single participant at 18 months of age. The corresponding atlas segmentation consists of 159 ROIs for the left and right hemispheres. Anatomically and functionally relevant major WM tracts were selected from the atlas (*n* = 59 ROIs) and grouped into the brainstem, projection, association, and commissural fiber tracts for further analysis (Table 3). The atlas segmentation was slightly manipulated by expanding the ventricles by two voxels. This was done because of the considerable enlargement of the ventricles, which was present in the majority of the patients with PMD.

**TABLE 3:** WM tract and corresponding JHU18m atlas ROI selection.

WM tract	ROI label name left and right of Jhu18m
Brainstem	Thalamus, midbrain, pons, cerebellum, middle cerebellar peduncle, medial lemniscus, medulla
Projection fibers	Anterior corona radiata, superior corona radiata, posterior corona radiata, posterior thalamic radiation (include optic radiation), anterior limb of internal capsule, posterior limb of internal capsule, retrolenticular part of internal capsule, cerebral peduncle (CST midbrain level), corticospinal tract (CST pontine level)
Association fibers	Superior longitudinal fasciculus, superior fronto‐occipital fasciculus, Inferior fronto‐occipital fasciculus, sagittal stratum, uncinate fasciculus, optic tract, cingulum, cingulate gyrus
Commissural fibers	Genu of corpus callosum, body of corpus callosum, splenium of corpus callosum, anterior commissure

### 2.6 Registration workflow

**FIGURE 1:**
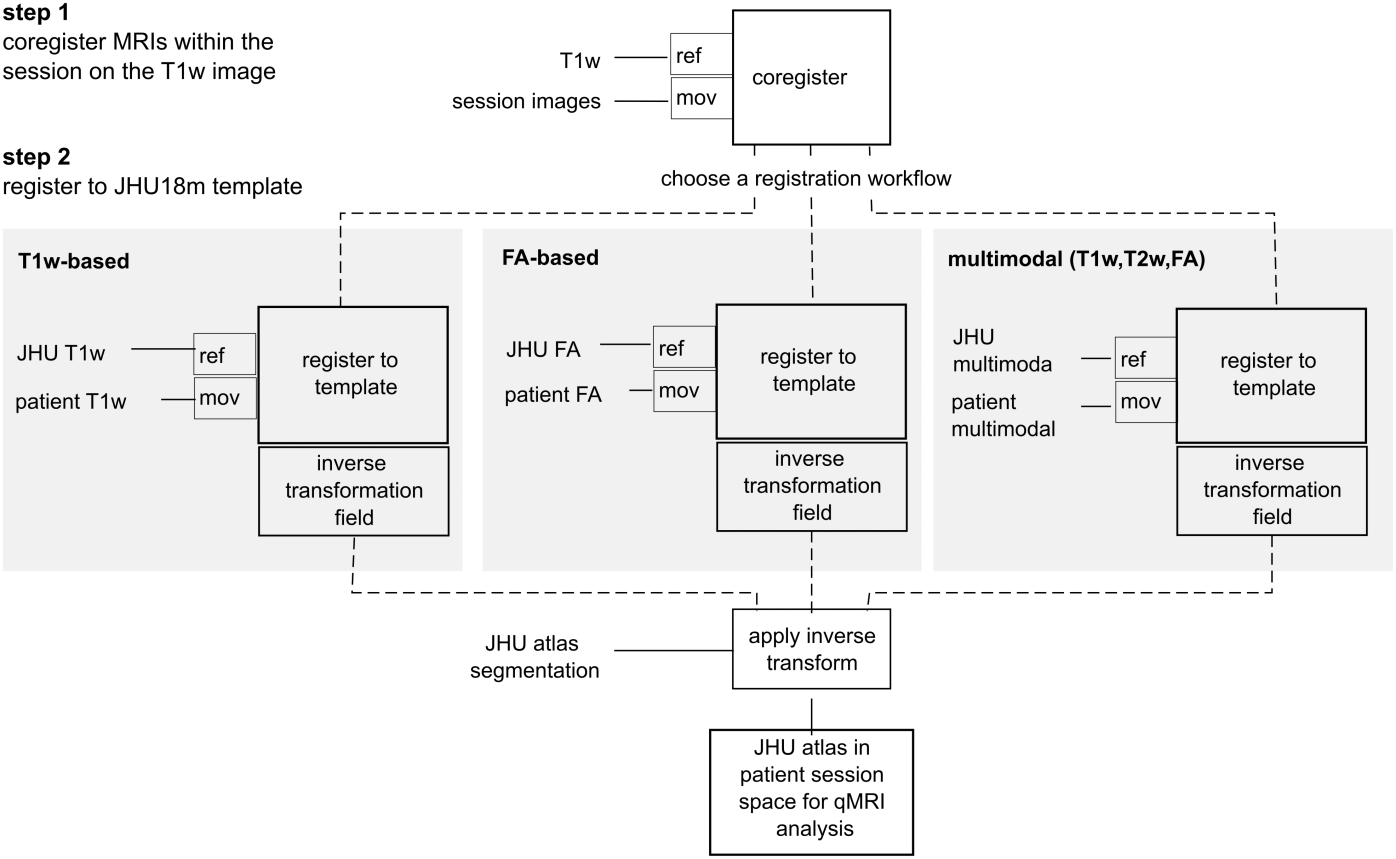
Schematic of the developed registration workflows; input images: mov: moving image; ref: reference image.

Nipype was also used to generate the postprocessing workflow. This workflow consisted of two steps shown in the flowchart (Figure 1). First, the T1w image was corrected for the bias field and all images within the session of a PMD patient were co‐registered to this image using *AntsRegistrationSyNQuick* transform type: “a” (affine) (Avants et al., 2011). Subsequently, the brain mask was extracted from the T1w image using FSL 5.0 BET and applied to the co‐registered images by using FSL math (ApplyMask). In the second step, three methods for registration to the template were implemented for PMD patients. In (a) the registration was based on the T1w image of PMD to a T1w template image, in (b) the FA image of PMD to an FA template image, and in (c) the multimodal images (T1w, T2w, and FA) of the PMD patient to the reference image(s) in the template. Thus, *AntsRegistrationSyNQuick* and transform type “s” (rigid + affine + deformable syn) were used. Final transformations were applied to transform the co‐registered images to the template to investigate registration accuracy, and the inverse transform was applied to the atlas segmentation for atlas‐based image quantification of qMRI data.

### 2.7 Assessment of registration accuracy

An experienced neuroradiologist (HHK) visually inspected the registration accuracy of all patients with PMD using three scoring categories: aligned, minor misaligned (mismatch ≤2 voxels), or major misaligned (mismatch >2 voxels). The evaluation compared the alignment of the registered images with the template images within five WM structures: corpus callosum truncus (body) and splenium, internal capsule, thalamus, and mesencephalon. A total of 25 ROIs (5 PMD × 5 ROIs) were scored to determine the registration accuracy.

Additionally, the registration accuracy was assessed using the structural similarity indexing method (SSIM) implemented in Python's *skimage.metrics.structural_similarity* (Wang et al., 2004). The SSIM map was generated to measure the spatial registration accuracy between the reference FA template image and the registered (transformed) PMD FA image, by identifying structural similarities and discrepancies. The SSIM algorithm assessed correlation loss, luminance distortion, and contrast distortion between two images using a sliding window approach. The obtained SSIM values ranged from −1 (*dissimilar*, *for example*, *misaligned*) to 1 (*similar*, *aligned*), thereby facilitating the estimation of spatial alignment and misalignment.

### 2.8 Atlas‐based image quantification of MWF in patients with PMD and healthy controls

To investigate differences in MWF between PMD and controls, the transformed atlas ROIs were superimposed on the MWF maps to quantify their mean and standard deviation, using FA‐based registration for PMD and conventional T1w‐based registration for controls.

### 2.9 Statistical analysis

Statistical analysis was conducted using the Python scipy.stats package. Differences in SSIM measures for various registration modalities were compared using a Student's *t* test for two independent samples with a significance level set at 0.05. Differences in MWF between healthy controls and patients with PMD were estimated using the nonparametric Mann–Whitney *U* test, with a significance level also set at 0.05.

## 3 RESULTS

**FIGURE 2:**
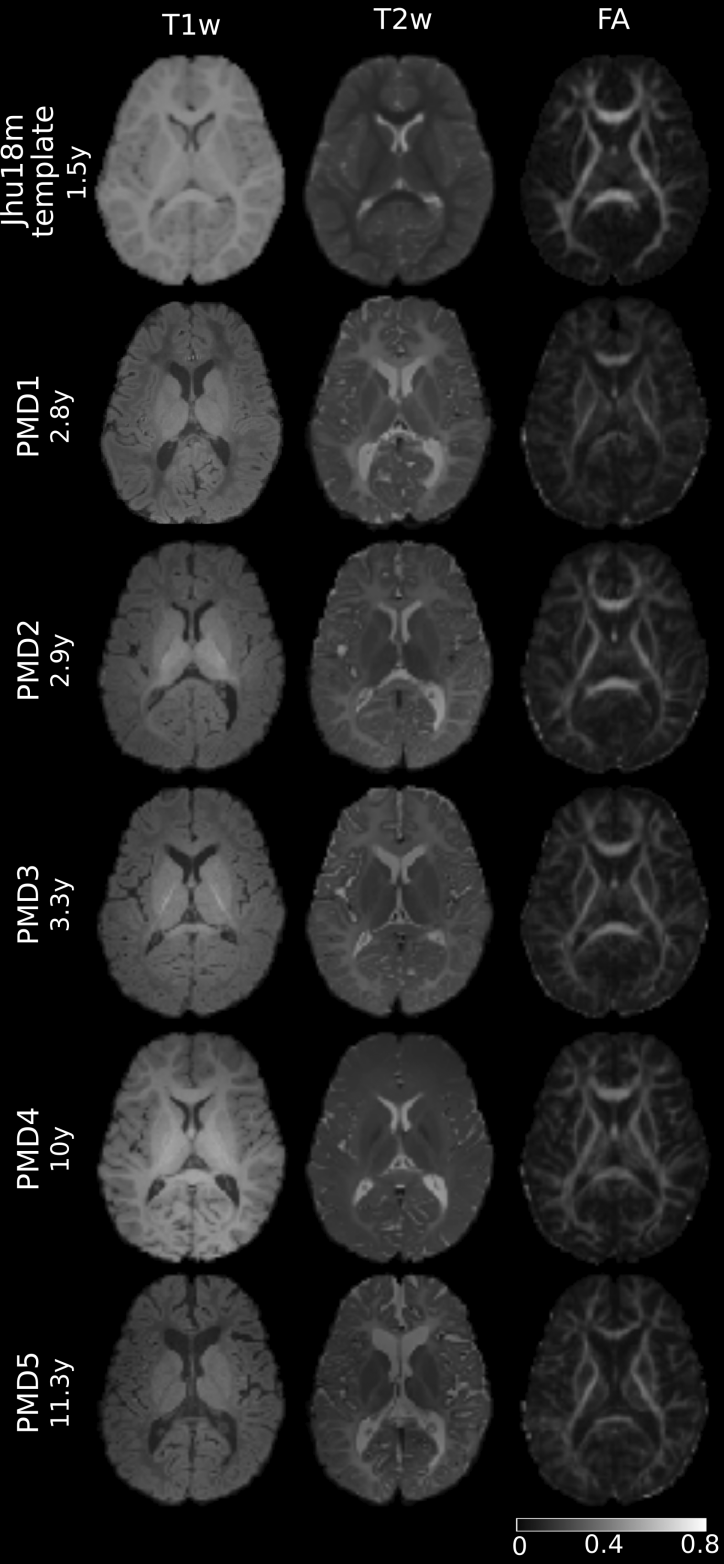
Cerebral axial T1w, T2w, and FA images of the registration dataset aligned to JHU18m template. Upper row: JHU18m template from a 1.5‐year‐old healthy participant. Rows 2–6: PMD patients of different ages, reflecting the inhomogeneous range of T1w and T2w tissue contrast, whereas FA‐image contrast was more similar in these hypomyelinated states with widely varying degrees of maturation.

This single‐center, retrospective study included five male patients with PMD (mean age, 6 years ± 3.8). MRI data were of good quality for further data analysis. In 4/5 of the patients, the WM appeared hypointense relative to the GM on T1w images, demonstrating the severely altered appearance compared to the healthy control template and abnormal myelination (Figure 2, left column). In addition, all T2w images indicated abnormal WM maturation as reflected by hyperintense to isointense signal presentation of the WM compared to the GM (Figure 2, middle column). Note that the tissue contrast of the FA images was more similar between the template and the patients with PMD, regardless of the different developmental stages of brain maturation (Figure 2, right column).

### 3.1 Assessment of registration accuracy for PMD participants

#### 3.1.1 Visual inspection

**FIGURE 3:**
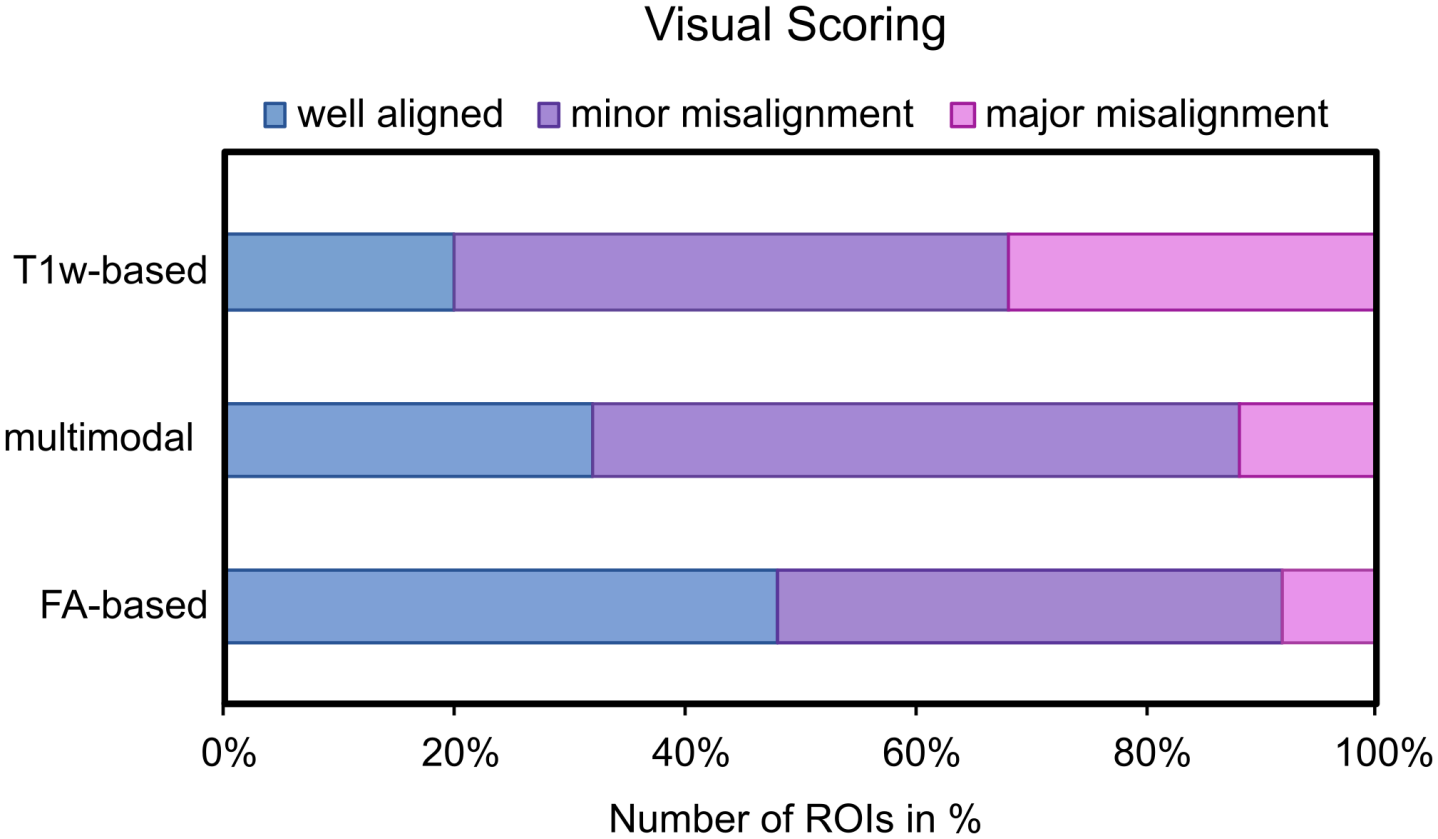
Visual scoring of registration accuracy for the three different image registration methods.

All patients were successfully registered to the JHU18m template. The registration accuracy ratings are presented in Figure 3 and indicated that FA‐based registration yielded the most accurately aligned brain structures and the fewest major misaligned ROIs. Misalignment was most pronounced in the T1w‐based registration, which also had the lowest percentage of aligned ROIs. Multimodal registration did not outperform FA‐based registration.

#### 3.1.2 Structural similarity evaluation

**FIGURE 4:**
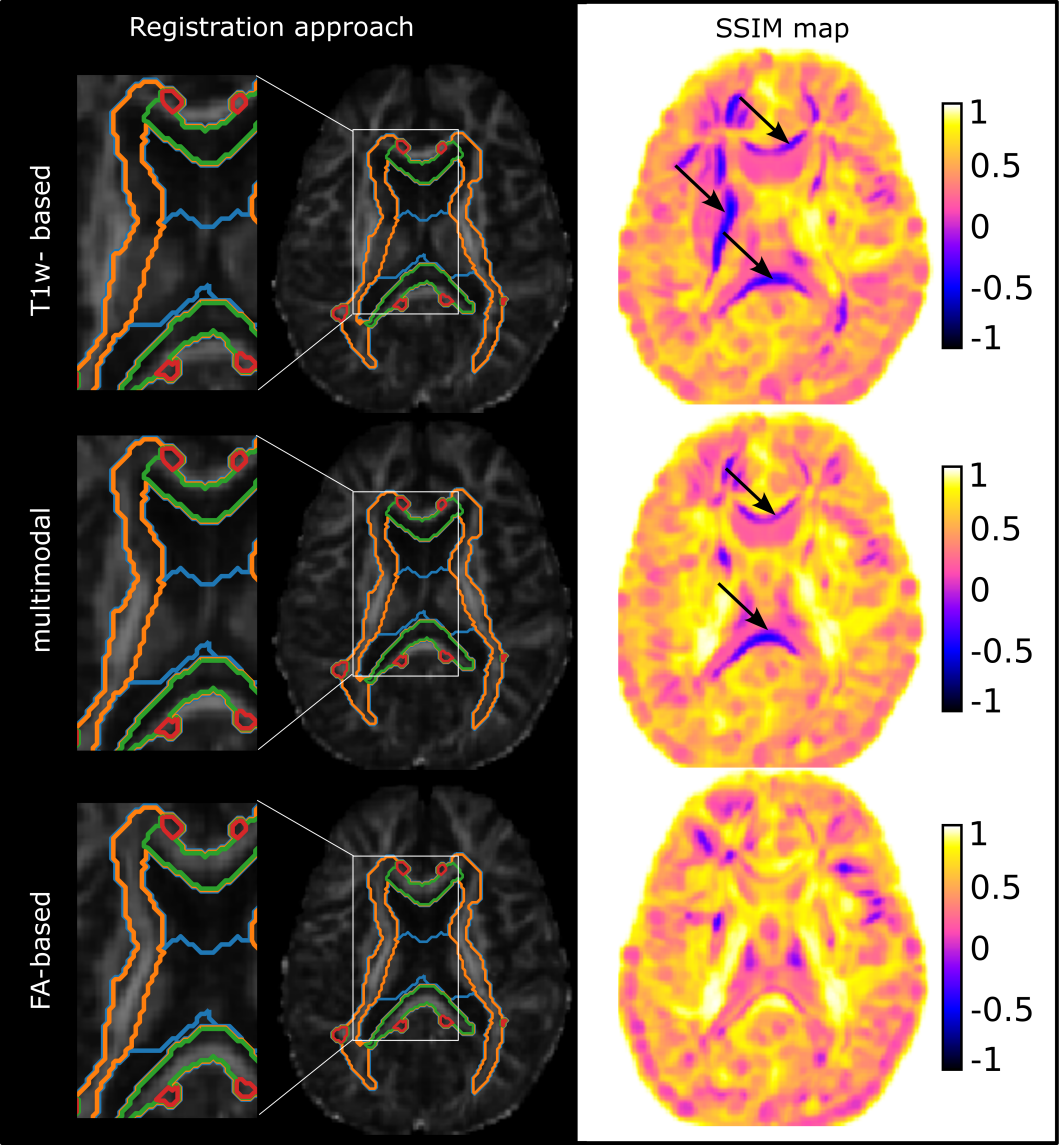
Transformed FA image of PMD5 in the JHU18m template space and zoomed section with superimposed atlas segmentation contours. The color‐coded SSIM map demonstrates the differing image similarities between the transformed FA images of the PMD patient to JHU18m for the three analyzed registration approaches. Major misaligned regions are indicated with black arrows.

To illustrate the differences in registration accuracy Figure 4, shows the transformed image, atlas segmentation contours, and SSIM maps for a registered patient with PMD in the template space. The lack of correspondence between the atlas contours and the WM tracts visualizes poor registration accuracy, which was associated with low SSIM values (arrows in Figure 4). The SSIM maps facilitated the evaluation of spatially misaligned areas, which were most evident for commissural fiber ROIs in T1w‐based registration. Table 4 shows SSIM values per WM tract for the different registration approaches. Mean SSIM values were highest for FA‐based registration, followed by multimodal and T1w‐based registration across all ROIs examined. Registration accuracy also differed significantly between T1w and multimodal registration, T1w and FA‐based registration, but not for multimodal and FA‐based registration, with *t*‐values *t* = −3.687 (*p* < 0.001), *t* = −5.531 (*p* < 0.001), and *t* = −1.570 (*p* = 0.125), respectively.

**TABLE 4:** Registration accuracy.

WM‐tracts	SSIM_T1w mean (SD)_	SSIM_mutimodal mean (SD)_	SSIM_FA mean (SD)_
Brain stem	0.55 (0.12)	0.60 (0.12)	0.62 (0.07)
Projection fibers	0.43 (0.09)	0.55 (0.10)	0.71 (0.08)
Commissural fibers	0.20 (0.19)	0.57 (0.30)	0.68 (0.24)
Association fibers	0.41 (0.07)	0.60 (0.10)	0.67 (0.10)
Mean (SD)	0.40 (0.14)	0.60 (0.03)	0.67 (0.04)

*Note*: SSIM values represented as mean (standard deviation) within atlas ROIs of the brainstem, projection, commissural, and association fibers using the three registration approaches.

Abbreviations: SSIM_T1w_, registration based on T1w; SSIM_multimodal_, registration based on T1w, T2w, and FA; SSIM_FA_, registration based on FA images.

### 3.2 Application of atlas‐based image quantification of MWF in patients with PMD and comparison with healthy controls

**FIGURE 5:**
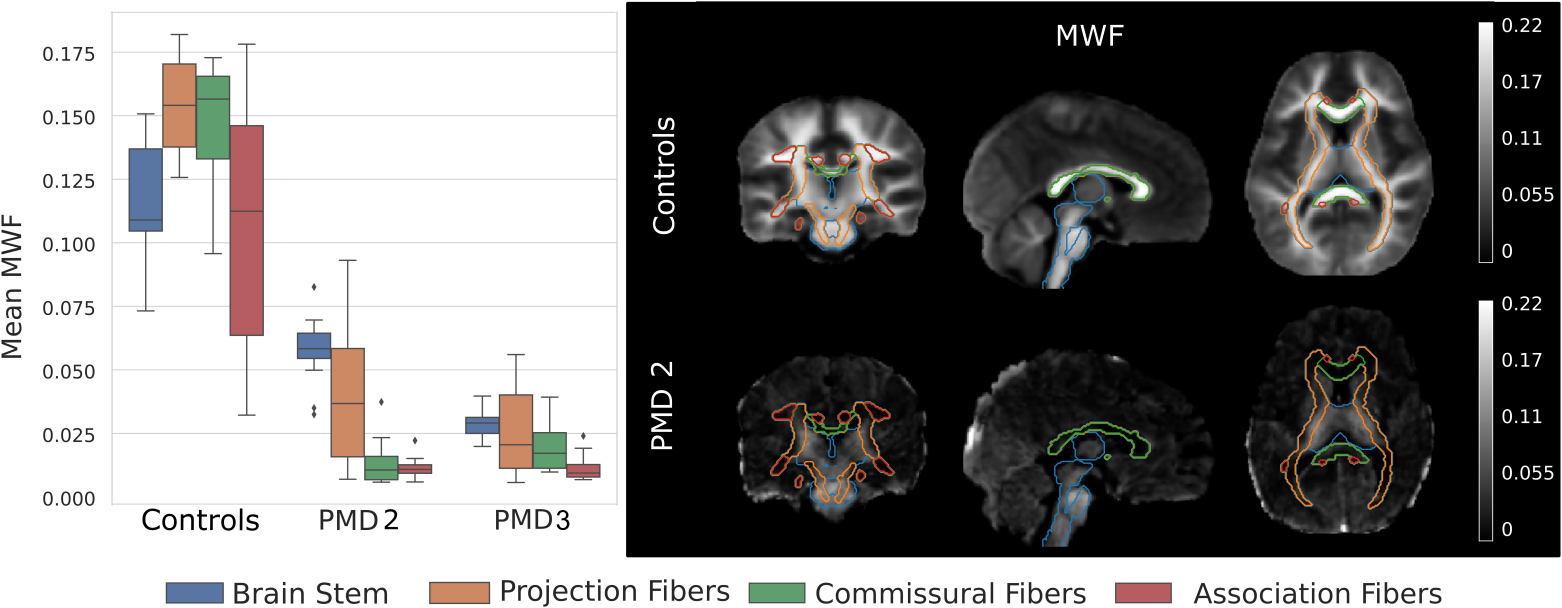
Left: Distribution of mean MWF within the ROIs grouped by the WM tracts for a 3‐year‐old control group and two patients with PMD at 2.9 and 3.3 years of age. Right: Corresponding MWF maps for the average image of the nine male controls and PMD 2 with superimposed WM tracts for comparison.

Based on the aforementioned findings, we selected the FA‐based registration approach to transfer the atlas segmentation to the patient space to evaluate MWF in patients with PMD, while T1w‐based registration was used for healthy controls. MWF was significantly lower within all selected WM ROIs in patients with PMD compared to age‐matched controls (mean MWF_PMD_ = 0.02672 ± 0.021 vs. mean MWF_Controls_ = 0.1299 ± 0.039, *p* < 0.05). To illustrate the spatial distribution of the MWF, Figure 5 shows the mean MWF, grouped by WM tracts, for healthy controls and patients at approximately 3 years of age. In controls, mean MWF in projection, commissural, and association fibers exceeded that of the brainstem. In contrast, in both PMD patients, the mean MWF was highest within the brainstem and some projection fiber ROIs, while the mean MWF of the commissural and association fiber ROIs was considerably lower than in the brainstem areas.

## 4 DISCUSSION

In hypomyelinating leukodystrophies such as PMD, MR signal intensity on conventional T1w and T2w images can be severely altered compared to a healthy brain due to myelin deficiency. This makes template registration a challenging task. Therefore, this study evaluated three registration approaches and their accuracy for registering PMD images to a pediatric healthy control template and applied the most accurate one for atlas‐based analysis of PMD qMRI data. The data and postprocessing pipelines created are publicly available via https://gitlab.ukdd.de/nra/wmi/atlas-based-assessment-of-hypomyelination.

Substantial differences in registration accuracy were found between the three registration approaches. The results showed that registration accuracy, as measured by visual inspection and SSIM values, was highest for FA‐based registration, followed by multimodal and T1w‐based registration. This result can be explained by the fact that template registration algorithms try to match image regions of similar intensity. The DTI‐derived contrast of the FA maps, which is mainly driven by the orientation and organization of the WM fibers, provided the most equivalent contrast for the template registration of patients with PMD. Another study similarly reported FA images to be superior for the analysis of very low‐contrast MRI images of adult patients with PMD (Al‐Saady et al., 2022). The inclusion of multimodal images in the registration process did not improve the registration accuracy in our study, despite the increased resolution and image information compared to FA images alone.

The analysis of SSIM maps offered the advantage of visualizing misaligned areas and computationally evaluating registration accuracy estimates. This may help select appropriate registration procedures for diseased brains, such as those with HLD. SSIM is useful for image quality assessment but is not yet widely used in medical image data analysis (Mudeng et al., 2022), for example, estimating the registration accuracy. Further studies are needed to demonstrate the utility of SSIM for detecting regional misalignment in a larger cohort. Nonetheless, SSIM has found its way into registration strategies (Larrey‐Ruiz et al., 2019; Sassi et al., 2008).

Our study demonstrated that atlas‐based qMRI image evaluation is also feasible in pediatric patients with PMD. In the current investigation, we evaluated MWF, which has been previously studied using the mcDESPOT approach for the in vivo assessment of normal brain myelination in early childhood (Deoni et al., 2012). This allowed us to compare normative MWF data from controls with patients with PMD and provided the opportunity to identify atypical myelin development. Mean MWF within main WM tracts was found to be significantly lower in PMD than in controls, reflecting a reduced or absent myelination. Specifically, MWF was present in brain structures that were myelinated at birth (brainstem) or shortly thereafter (corona radiata). Surprisingly, MWF was barely detectable in other brain regions such as commissural and association fibers in patients with PMD. In contrast, in healthy controls following physiological progressing myelination mean MWF in projection, commissural, and association fibers exceeds that of the brainstem at 3 years of age. Thus, these findings provide new insights into spatial myelin deficits and a delayed or disturbed pattern of myelination in the patients with PMD studied. Our findings were consistent with mcDESPOT results from a preclinical model of PMD, the shaking pup canine model, which suffers from hypomyelination showing a profound lack of MWF in the WM compared to controls (Hurley et al., 2010).

However, this study had limitations. First, the limited availability of PMD imaging data due to its ultra‐rare nature. Second, the small number of pediatric brain templates that also included an FA map and detailed atlas segmentation. The choice of a single‐subject template was a necessary compromise for the use of near‐age‐appropriate anatomical brain structures, with the trade‐off for less variation in anatomy. Third, FA‐based registration also had some limitations. For example, FA‐based registration is mainly driven by the high signal intensity of the major WM tract structures, reflecting axonal integrity and density (Harsan et al., 2006; Song et al., 2002), but lacks contrast in brain regions such as the ventricle/WM or WM/GM tissue boundaries, which in turn may lead to poorer registration accuracy in these regions. In order to address this issue, it could be considered to use additional image information from the other DTI metrics (MD, AD, RD). However, axonal involvement is present in patients with PMD albeit to varying degrees depending on the genetic defect and age of the patient (Laukka et al., 2014; Sima et al., 2009). Fourth, another challenge in the registration of PMD data was the substantial atrophy of brain structures such as the corpus callosum (CC) in some of the patients. Reduced CC area in patients with PMD has been previously described (Laukka et al., 2013), as well as CC atrophy in a longitudinal MRI study (Sarret et al., 2016). The greater the regional atrophy, the more difficult it was to achieve registration accuracy to a healthy template. That is because the registration algorithm used restricts the deformation by considering the deformation of neighboring tissue structures. This may result in a slight discrepancy between the registered PMD imaging data and the atlas segmentation. For the registration approach performed in this study, we used antsRegistrationSyNQuick, which uses robust fixed parameters. However, further improvements may be beneficial with the more flexible antsRegistration algorithm, such as the manipulation of the cost function or the regularization, the latter being a factor that constrains the transformation between the moving and fixed images. Fifth, we did not explore other likewise available neuroimaging tools like SPM and FSL, to register the PMD data or investigate the registration accuracy in other HLD imaging data. This was beyond the scope of this study.

Future research might focus on comparison with other qMRI imaging biomarkers to determine different aspects of microstructural tissue change in patients with PMD. Furthermore, the heterogeneity and temporal evolution of myelination in these patients are poorly understood and need to be addressed in future studies.

## 5 CONCLUSION

FA‐based template registration is superior to conventional T1w or multimodal registration in patients with PMD and provides an alternative approach for the successful evaluation of myelination using qMRI in pediatric patients with hypomyelinating leukodystrophies. The atlas‐based analysis of MWF revealed atypical WM myelination for patients with PMD in comparison to controls and allowed quantification of its extent. The developed postprocessing pipeline may be supportive of investigating further potential biomarkers to assess spatial myelin deficits in childhood WM disorders including HLD.

## AUTHOR CONTRIBUTIONS

**Caroline Köhler**: Postprocessing; formal analysis; writing original draft; funding acquisition. **Paul Kuntke**: McDespot processing; data curation. **Prativa Sahoo**: DTI processing; data curation. **Hannes Wahl**: Data curation. **Sean C. L. Deoni**: Supervision. **Jutta Gärtner**: Supervision; patient recruitment. **Steffi Dreha‐Kulaczewski**: Patient recruitment; data acquisition; conceptualization; supervision; funding acquisition. **Hagen H. Kitzler**: Conceptualization; supervision; visual inspection; funding acquisition.

## CONFLICT OF INTEREST STATEMENT

The authors declare no conflicts of interest.

## Data Availability

The data presented in this study are openly available at https://gitlab.ukdd.de/nra/wmi/atlas-based-assessment-of-hypomyelination. The pseudonymized MRI data are organized according to the Brain Imaging Data Structure standard. The quantitative MRI data and registration results are available at https://gitlab.ukdd.de/nra/wmi/atlas-based-assessment-of-hypomyelination/derivatives. The Python code developed for the registration pipelines and the estimation of structural similarity index are available as Jupyter notebooks at https://gitlab.ukdd.de/nra/wmi/atlas-based-assessment-of-hypomyelination/code. We have the permission to use a pediatric healthy control dataset published by Deoni et al. (2012).
